# Pheromones, binding proteins, and olfactory systems in the pig (*Sus scrofa*): An updated review

**DOI:** 10.3389/fvets.2022.989409

**Published:** 2022-12-01

**Authors:** Devaraj Sankarganesh, Roy N. Kirkwood, Patricia Nagnan-Le Meillour, Jayaraman Angayarkanni, Shanmugam Achiraman, Govindaraju Archunan

**Affiliations:** ^1^Department of Microbial Biotechnology, Bharathiar University, Coimbatore, India; ^2^Department of Biotechnology, School of BioSciences and Technology, Vellore Institute of Technology, Vellore, India; ^3^School of Animal and Veterinary Sciences, The University of Adelaide, Roseworthy, SA, Australia; ^4^University Lille, CNRS, USC INRA 1409 - UGSF - Unité de Glycobiologie Structurale et Fonctionnelle, Lille, France; ^5^Department of Environmental Biotechnology, Bharathidasan University, Tiruchirappalli, India; ^6^Department of Animal Science, Bharathidasan University, Tiruchirappalli, India

**Keywords:** chemical signaling, olfaction, steroid pheromones, vomeronasal organ, olfactory receptor (OR)

## Abstract

Pigs utilize multimodal communication for reproductive and other behaviors, and chemical communication is one of the key components. The success of reproduction relies on chemical communication favored by the steroid pheromones from boar saliva. These steroids were proven to be involved in advancing puberty in gilts (the boar effect) and in promoting estrus behaviors in gilts/sows, thereby helping to detect estrus and facilitating the timing of artificial insemination. The steroid pheromones bound with carrier proteins are evidenced in the mandibular (submandibular) salivary secretions of the boar. These salivary steroids bind with carrier proteins in the nasal mucus and vomeronasal organ (VNO) of the sows, eventually triggering a cascade of activities at the olfactory and endocrine levels. Besides steroid pheromones, pig appeasing pheromones (from mammary skin secretions of sows) have also been demonstrated to bind with carrier proteins in the nasal mucus and VNO of the piglets. Thus far, four different proteins have been identified and confirmed in the nasal mucus and VNO of pigs, including odorant binding proteins (OBPs), salivary lipocalin (SAL), pheromaxein, and Von Ebner's Gland Protein (VEGP). The critical roles of the chemosensory systems, main olfactory systems and VNO, have been comprehensively reported for pigs. This review summarizes the current knowledge on pheromones, their receptor proteins, and the olfactory systems of porcine species.

## Introduction

Pheromonal signals in pigs are perceived and processed through various components, in that binding protein and the olfactory systems [main olfactory system (MOS) and Vomeronasal organ (VNO)] are pivotal. The binding proteins and olfactory systems were extensively investigated during the past three decades. In particular, molecular studies revealed novel binding proteins and elucidated the structural and functional aspects of the olfactory systems. Put together, these research results improved our understanding of pig chemical communication; however, a comprehensive and updated review on these topics is yet to be available. While we recently reviewed the pheromonal communication of pigs (1), in this review we intend to comprehend the facets of the chemical communication system, with a special focus on carrier proteins and olfactory systems.

Pheromones are species-specific chemical moieties secreted in various body fluids that elicit specific behavioral and/or neuroendocrinological changes in the receiving individual of the same species. In many animals, various body secretions such as urine, feces, saliva, glandular secretions, and tears have been shown to be sources of pheromones. In pigs, boar saliva and sow mammary skin secretions have been documented as key sources of pheromones. Saliva contains steroid pheromones (sex pheromones; androstenone and androstenol) that induce the boar effect in gilts, i.e., advancing the onset of puberty. In common with rodents, an immediate increase in LH pulse frequency was seen in prepubertal gilts exposed to a boar, but only in those destined to show an earlier puberty (2). An increased level of oxytocin was found in a higher percentage of sows that also showed a longer standing response. It is apparent that oxytocin facilitates the expression of receptive behaviors in sows in coordination with other neuroendocrine hormones. However, the auditory and tactile stimuli of boars did not induce an increase in oxytocin levels, rather the presence of a live boar induced the effect. Therefore, the presence of live boar was indeed essential in regulating the boar effect (3).

Boar salivary steroid pheromones were proven to induce estrus behaviors in sows and so help to detect estrus. Unlike boars, there are no reports of salivary pheromones in sows although non-steroid pheromones were reported in their mammary skin secretion. Briefly, these non-steroid pheromones (a mixture of fatty acids) potentially reduce aggression between the piglets. In addition to the steroid pheromones, saliva contains specific proteins that are believed to act as carrier molecules for the steroid pheromones.

## Pheromones and pheromonal effects in pigs

**Figure 1:**
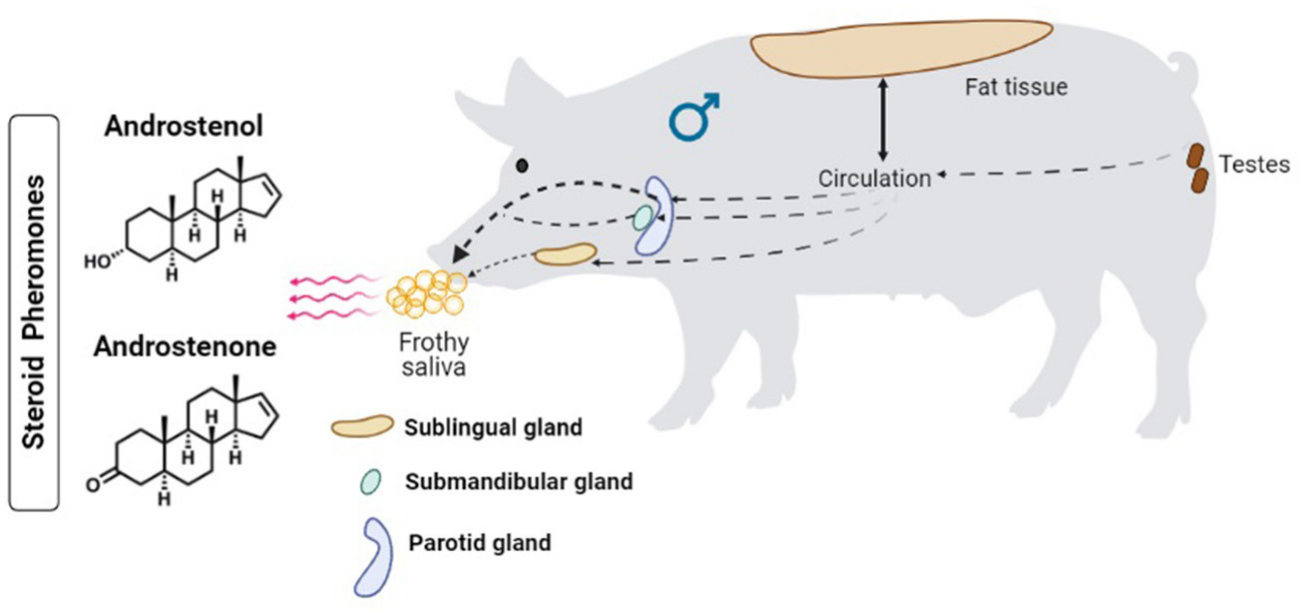
Mechanism of secretion and excretion of steroid pheromones from boars.

Boar salivary glands, principally the mandibular (submandibular) gland, secrete the saliva that carries the steroid pheromones. These pheromones are primarily produced in the testes, transported *via* the blood circulatory system, stored in adipose tissues, and in part secreted through the saliva (Figure 1). The primary salivary steroids were identified as androstenone and androstenol, and sow behavior assays suggested that these pheromones successfully elicited estrus behavior (4). In the salivary gland, 16-androstenes and the corresponding enzymes that are responsible for the double bond formation at C-16 and C-17 were absent (5). These authors confirmed the secretion of androstadienol in the testes and subsequent transportation to the mandibular gland. They further suggested the presence of 5-α-androstenone and 3-α- and 3-β-androstenols in the mandibular gland was favored by transportation from the blood circulatory system. Interestingly, Gower et al. (6) reported that 3α-hydroxysteroid dehydrogenase and 3β-hydroxysteroid dehydrogenase were essential to convert androstenone to 3α-androstenol and 3β-androstenol, respectively. However, Katkov et al. (5) reported the absence of 3α-hydroxysteroid dehydrogenase in the salivary gland of boars, while the primary compounds such as pregnenolone and progesterone were found only in the testes of boars where metabolism occurs to derive other androstenes (7). Nevertheless, the occurrence of steroids in the mandibular gland was comparatively lower than in the testes. For instance, the boar saliva was evidenced to contain many steroids including 3α-androstenol and 5α-androstenone (8), and 3β-androstenol (9), while the mandibular gland was found to contain only trace amounts of androstadienone and 17β-hydroxy-5α-androstan-3-one (5, 10). These odorous steroids were implicated in the boar effect.

Recently, a synthetic mixture including androstenone, androstenol, and quinoline as a boar saliva analog was shown to elicit estrus behaviors in sows and, potentially, to reduce their weaning-to-estrus intervals (11), and to increase the number of pigs born per litter (12). The habituation and dishabituation paradigm proved that >0.80 ppm of boar saliva analog was required to be perceived by the gilts (13). Although this boar saliva analog has been found to elicit comparable sow behavioral responses to those of steroid pheromones, evidence is lacking for the presence of quinoline in boar saliva. Therefore, confirmation of quinoline in boar saliva is needed and its role in the pheromonal communication of pigs verified. Considering the available literature on pig pheromones, we recently suggested that pigs use multimodal communication for reproduction in that chemical, visual, auditory, and tactile communication are important (1). Chemical communication may play a significant role but a synergistic effect with other communication modalities cannot be ruled out.

In addition to steroid sex pheromones, non-steroid pheromones (pig appeasing pheromones; PAP) were identified in the mammary skin secretions of suckling sows (14, 15). Exposure to PAP has been shown to modify the beneficial and aversive behaviors among weaned piglets but at variable levels based on the setup of the studies (15, 16). The components (a cocktail of fatty acids) of PAP were demonstrated to bind with various carrier proteins in pig nasal mucus, thereby confirming their potential role in the olfactory and vomeronasal systems (17). Field-level studies indicated that PAP produced variable effects on piglets by reducing the negative social behavior (18, 19).

## Components of olfactory communication in pigs

Pigs rely heavily on their sense of smell and are an excellent model system to investigate olfaction (20). The olfactory bulb and the olfactory nerve layer of the pigs contain axons that are projected from the olfactory sensory neurons of the olfactory mucosa. The olfactory bulbs contain 11,000 glomeruli with considerable size variation (21). The olfactory structures of pigs do not possess any unusual organization and the olfactory bulb and olfactory cortices are similar to other mammalian species, indicating a possible lineage among mammals in olfaction (21). However, Nguyen et al. (20) identified 1,301 olfactory receptor (OR) related sequences in pigs, of which, 1,113 were likely to be functional. In contrast, 636 genes identified in humans, out of which, 339 are intact and likely to encode functional odorant receptors. This indicates the superior olfactory capacity and functional diversity of the olfactory system of pigs.

The detailed analysis of sensory systems in other animals led to the discovery of the Grueneberg ganglion that expresses odorant olfactory receptors. Grueneberg ganglia are involved in the detection of odors and alarm pheromones (22). Further, the septal organ in the nasal septum has been reported to respond to odorants (23). However, neither Grueneberg ganglia nor the septal organ have been identified in pigs.

## Pheromone binding proteins

Many soluble binding proteins are present in the body exudates and olfactory systems of animals to deliver and receive volatile pheromones (24). For instance, the urine of mice and rats contains major urinary proteins (MUPs) and alpha 2-u globulin, respectively, which carry volatile ligands or pheromones (25–27). In mice, volatile pheromones (3,4-dehydro-Exo-brevicomin and 2-sec-butyl-4,5-dihydrothiazole) were identified as bound ligands within the urinary carrier proteins (26). Later studies also attested the presence of bound ligands and elucidated their active role as pheromones in puberty acceleration of female mice (28). The key notion is that these bound ligands are present in the urinary proteins of male mice and regulate the reproductive and developmental aspects of female mice. The MUPs devoid of bound ligands carry pheromonal properties. These MUPs accelerate puberty onset in female mice (29), and the bound volatiles co-eluted with MUP attract females (30).

The VNO and nasal mucosa of both boar and sow contain odorant binding proteins (OBPs), Von Ebner's Gland Protein (VEGP), and salivary lipocalin (SAL). In addition, SAL and pheromaxein were reported in the mandibular gland of boars. Bound ligands (5α-androst-16-en-3-one and 5α-androst-16-en-3α-ol) were also identified in the sex-specific binding proteins of the mandibular gland of adult boar (31). However, the pheromonal properties of OBPs, pheromaxein, VEGP, and SAL have not been evaluated in isolation and it remains unknown whether the carrier protein devoid of ligands retain pheromonal properties.

### Proteins in boar mandibular glands (pheromaxein and SAL)

The mandibular gland in boars is the key organ in the secretion of frothy saliva that contains active steroid pheromones and induces sexual behaviors in sows. The presence of two different carrier proteins (SAL and pheromaxein) in the mandibular gland with similar functions makes pig an interesting chemosensory research model. Even though SAL and pheromaxein belong to different protein families (lipocalin and secretoglobin, respectively), both revealed steroid pheromones as bound ligands, suggesting an indispensable role in the chemical communication of pigs. Hitherto, the presence of SAL and pheromaxein in the mandibular gland has been reported by separate studies, and no report for both the proteins in a single study is available. The two proteins may exhibit differential binding affinity toward steroids and so comparative analysis of SAL and pheromaxein in the same boar is recommended, which may shed light on pheromonal communication in pigs.

The 16-androstene binding protein, pheromaxein, was identified in the saliva and mandibular gland of Gottingen miniature pigs (32). Subsequently, Booth and von Glos (33) confirmed pheromaxein in the mandibular gland of boars of different breeds, which was in higher amounts than other proteins synthesized. The amount of pheromaxein was lower in the parotid gland, corresponding to the level of steroids in the respective salivary glands. Babol et al. (34) similarly reported pheromaxein in the salivary gland of boars and that its concentration was related to the 16-androstene steroid content. Further, they found a stable bond between pheromaxein and 16-androstene steroids that implied 16-androstene steroids were functional molecules in pig communication. Austin et al. (35) attested the presence of pheromaxein with bound ligands (androstenone and androstenol) in the mandibular gland of boars.

Besides pheromaxein, a large amount of a binding protein that possessed a lipocalin signature (-G-X-W-) was found in the mandibular gland of mature boars. This protein of molecular mass of approximately 22 kDa was identified as SAL (31). Unlike pheromaxein, which exists in three isoforms (A, B, and C), SAL exists in two isoforms. The two isoforms of SAL share sequence similarities with other lipocalins but differ in three amino acids. SAL also possesses steroidal compounds as natural ligands. Interestingly, competitive binding assays revealed that androstenone efficiently binds with both isoforms of SAL demonstrated by replacement of the fluorescent probe, 1-aminoanthracene. SAL displayed an affinity toward the steroid pheromones (androstenone and androstenol) but not toward other small molecules. It is perhaps due to the reduced Van der Waals bonding between the compounds (small molecules) and the wall of the protein, as the binding cavity of SAL is larger than other binding proteins (36). Given the binding specificity to pheromones and the similarity to urinary and salivary proteins of rodents, it has been proposed that SAL may itself act as a pheromone similar to MUP in mice (31), but this suggestion has not been examined.

The porcine OBP was identified as a dimer with a β-barrel structure, which is formed of aromatic or aliphatic chains in addition to a few polar non-charged residues. This β barrel structure is linked to an α-helical domain, but there were no conformational changes upon binding with the odorants (37). The ligand-linked alterations of OBP dynamics, for instance, odorant-loaded OBP, could interact with OR or OBP-OR complex to facilitate the binding of the odorants. Subsequently, the odorant could be released in the medium, which could further activate OR, which possibly results in the signal transduction. Altogether, the involvement of various elements facilitate the chemoreception in pigs (38, 39).

### Proteins in nasal mucus and VNO (OBP, VEGP, and SAL)

Hancock et al. (40) tested the steroid-binding efficiency of olfactory and respiratory tissues and found increased uptake of androstenone in olfactory tissue extracts. However, these authors omitted the mucosa of the VNO from their study which led to the speculation that VNO could also uptake androstenone. A later study revealed isoforms of additional lipocalins (OBP-II and OBP-III) that resemble Von Ebner's Gland Proteins (VEGP) and SAL structure in the nasal mucus of both sexes of pigs. These identified lipocalins were classified as odorant-binding proteins due to their inherent binding property with various ligands and the absence of endogenous ligands (41). Interestingly, OBP-III possessed amino acid sequence, binding properties, and an oxidation state similar to SAL, presumably indicating a possible role of OBP-III in binding with steroids and involvement in pheromonal communication by transferring the steroids into the neurons of VNO. However, these authors (41) did not study the carrier proteins in VNO. A later study with prepubertal pigs revealed OBP, VEGP, and SAL proteins in both nasal mucus and VNO (17). The binding affinity toward appeasing and sex pheromones of OBP and VEGP was evaluated by an in-gel binding assay. The OBP showed greater affinity toward palmitic and oleic acids, which are the major components of PAP. Two isoforms of VEGP were distinguished by their binding affinity: isoform 1 binds fatty acids of PAP, but isoform 2 binds only the steroid, progesterone (17). This interesting specificity was explained by the identification of the natural ligands of these isoforms (42). The ligand of VEGP isoform 1 was identified as testosterone, those of isoform 2 as fatty acids. The difference between the two isoforms is the presence of a single sugar, N-acetylglucosamine, on isoform 1.

**Figure 2:**
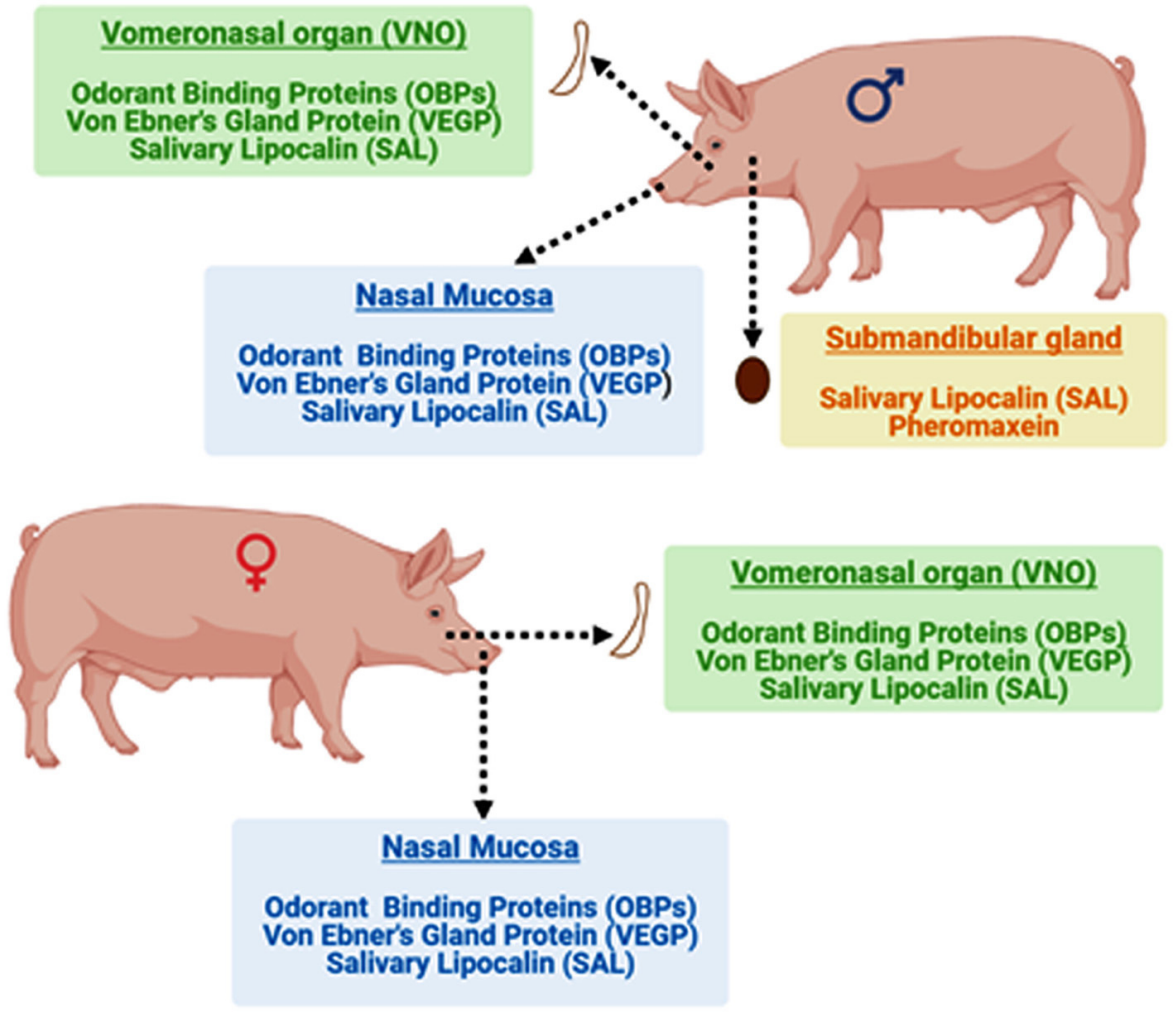
Localization of different glands the occurrence of various binding proteins that facilitate chemical communication in boars and sows.

The binding proteins identified in boars and sows are illustrated in Figure 2. The presence of different types of binding proteins in the nasal mucus and VNO suggests the involvement of both the main and the accessory olfactory systems in detecting and discriminating different pheromones (appeasing and sex pheromones) in pigs (17). OBP may act as a primary carrier in receiving the steroid pheromones and then transferring the stimulus to the VNO (43). However, the presence of SAL in the VNO raises the possibility that VNO could directly participate in receiving the steroid pheromones. Also, the synergistic involvement of both systems by involving both proteins cannot be discounted. However, it is still intriguing which tissue, nasal mucus or VNO, is primarily involved in receiving the steroid pheromones. Therefore, deciphering the primary receiver or synergistic effect is required to fully understand the importance of this particular subset of tissue and proteins. Above all, OR7D4, a seven transmembrane receptor is reported in horses and humans, which is exclusively binds to the androstenone (44). However, OR7D4 is hitherto not reported in boars and sows (45).

### Post-translational modifications of OBP, VEGP, and SAL

OBP and VEGP N-terminal sequences are characterized by the cyclization of the first residue Glu1 in pyroglutamic acid (Gln1) that makes the protein refractory to Edman sequencing (46). The N-terminus of SAL is His1, thus not affected by this modification. In addition, SAL is modified by N-glycosylation, a well-known post-translational modification of secreted proteins (31, 47). Complex N-glycan chains were identified on Asp53 (47). Recombinant SAL was produced by *E. coli* and was used together with native SAL purified from mandibular glands in fluorescent-based binding assays and the two proteins displayed comparable affinities for the tested ligands. This was surprising because native SAL bears N-glycans that are absent from the bacterial recombinant counterpart. Indeed, glycosylation is known to modify the function of the proteins. Such a result could indicate that SAL N-glycosylation is not involved in the internal binding of ligands, but more likely in interactions at the surface of the protein, such as dimer formation or interaction with olfactory receptors.

Phosphorylation is an important post-translational modification that also regulates the function of proteins. Phosphorylation, first identified in pig OBP, generates different isoforms with different binding affinities toward certain fatty acids of the PAP and steroid pheromones (48), supporting the reception of both appeasing pheromones and sex pheromones through OBPs. Nagnan-Le Meillour et al. (43) reported nine isoforms for VEGP, seven for SAL, and 12 for OBP in the nasal mucus of pigs resulting from post-translational modifications. Subsequent studies revealed that pig olfactory proteins were modified by two post-translational modifications, phosphorylation and *O*-N-acetylglucosaminylation (*O*-GlcNAcylation) (42, 49). In comparing the effect of these modifications, binding assays were performed with recombinant OBP isoforms, which were phosphorylated and native OBP isoforms (purified from nasal mucus), both of which were phosphorylated, and O-GlcNAcylated. Recombinant isoforms displayed variable affinity toward fatty acids of PAP and sex pheromones (48) whilst the native isoforms showed opposite binding specificity for either PAP or sex pheromones (50). Mapping of phosphorylation and O-GlcNAcylation sites by CID-nanoLC-MS/MS has allowed localization of phosphosites at S13 and T122, and HexNAc sites at S13 and S19. Taken together, the post-translational modifications appear to be a critical step in the determination of the binding specificity of OBPs (50). Indeed, some isoforms purified from nasal mucus have been shown to be tuned to the binding with androstenol and androstenone, which are part of the sex pheromone in boar saliva. This denotes a specialization of OBP isoforms in the molecular coding of pheromone components and suggests a fine regulation in the detection of the pheromone at different times of the pig life. The boar pheromone is perceived as a submission signal by male piglets and as an aphrodisiac by the mature female. Even if this difference in the behavior results from a different central processing in the cortex, the regulation of pheromone binding by post-translational modifications as soon as the perireceptor events of reception cannot be excluded. Thus, the control of behavior could be upregulated at every step of the olfactory system.

It is apparent from the above literature that pigs use different soluble proteins to carry/release and to receive various ligands, including steroid pheromones and fatty acids of PAP. However, the presence of the same proteins in the nasal mucus and VNO of both sexes is intriguing and suggests functions of the proteins in the chemical communication of pigs. The sex-specific expression and presence of pheromaxein in boars and their bound ligands confirm the pivotal role of these proteins in carrying the steroid pheromones to the receiver.

### Boar pheromone signaling in the olfactory subsystem

MacLeod et al. (51) revealed altered unit activity of olfactory responses in boars, castrated boars, and diestrus sows in response to 17β-hydroxy-4-androsten-3-one and suggested the presence of neurons in the olfactory bulb that were non-responsive, non-discriminatory, or discriminatory. Contemporaneously, Ellendorff et al. (52) tested 5α-androstenone and other steroid hormones in aerosols and observed unit activity in the mitral cell layer of the olfactory bulb, which is connected to the lateral olfactory tract and amygdala. In common with MacLeod et al. (51), the response was observed in boars (intact and castrated) and sows (diestrus stage), which led to the speculation that pheromone-responding neurons exist in pigs irrespective of their sexual and gonadal status. Subsequently, the electro-olfactogram responses revealed that 5α-androstenone could be detected by sows and boars (castrated), and threshold responses were as low as 10 ng (53). In contrast, Dorries et al. (54) reported differential responses to androstenone in boars and sows, with the threshold being one-half log unit lower in sows compared to boars; however, the responses to general odorants did not differ between boars and sows. This latter study thereby attested to the influence of sex in the functional valence of the olfactory systems and suggests a possible influence of hormonal milieu on the spatial pattern of the olfactory system.

The VNO is crucial for chemical communication in many animals. It connects to both nasal and oral mucus, thereby facilitating the effective transfer of odorant molecules. In order to demonstrate the importance of VNO in the detection of pheromone, Dorries et al. (55) used surgical cement to create VNO-intact and VNO-blocked sows and demonstrated that the sows did not show any difference in their attraction to either androstenone or mineral oil and concluded that detection of androstenone was not mediated through the VNO. Interestingly, Booth and Webb (56) reported that in goat does, cautery of the VNO completely blocked estrus behavior in response to the presence of bucks. More recently, Kondoh et al. (57) demonstrated type 1 VNO receptors in cattle and pigs, with similar documentation for other artiodactyl species including sheep and goats. Therefore, it seems likely that the VNO of pigs is functional and, while speculative, the conclusion of Dories et al. (55) may have been due to a failure to completely block the VNO.

In common with rodents, gilts demonstrate acceleration of puberty when exposed to boars (Vandenbergh effect) as well as increased variability of estrous cycle length when maintained in all-gilt groups (Lee Boot effect). However, unlike in rodents, only a few studies have reported the molecular components of olfactory system and VNO in pigs. Nguyen et al. (20) reported diverse olfactory receptor genes that included 1,113 olfactory receptor sequences in pigs. These diverse receptors were attributed to the functional difference of olfactory receptors in binding with various odorants and imply a sophisticated olfactory system in pigs. In parallel, Dinka et al. (58) reported 25 V1R genes, 10 of which were functional. The allelic diversity of V1Rs in pigs was less than in other animals.

Stefanczyk-Krzymowska et al. (59) used tritium-labeled androstenol to study its accumulation pattern in selected areas of the olfactory system (neurohypophysis, adenohypophysis, and ventromedial hypothalamus) and found it in the nasal mucus and cavity, whereby it could then be transported to the hypophysis. This may culminate in specific behavioral changes that occur in sows during boar exposure.

The mechanisms involved remain to be fully elucidated but as detailed by Stefanczyk-Krzymowska et al. (60), 5α-androstenol can cross the olfactory mucosa and be transported *via* blood circulatory system to various brain areas and the hypothalamus, bypassing the systemic circulation and avoiding hepatic clearance. Further, repeated intramuscular injections of this steroid into cycling follicular phase gilts resulted in reduced mean plasma LH and estradiol concentrations (59), reflecting either a negative feedback or a chronic stimulation depleting pituitary LH stores. While a serial steroid injection does not accurately reflect normal physiology, these results do support a possible humoral route for pheromonal transfer from the nasal cavity to the hypothalamus. Although these latter authors did not determine specific hypothalamic targets, an effect at the arcuate nucleus is implied by the effects on circulating LH concentrations, and by extension estrogen production. The arcuate nucleus contains Kisspeptin, neurokinin B, and dynorphin (KNDy) neurons that are the controllers of the GnRH pulse generator *via* kisspeptin release with more than 90% of the KNDy neuron afferents originating from within the arcuate nucleus (61). Further, GABAergic neurons adjacent to GnRH neurons within the arcuate nucleus have been shown to stimulate LH release (62), and androstenol is a neurosteroid that is a positive modulator of hypothalamic GABA receptors (63). Steroid modulation of GABA receptors has been reviewed previously (64).

Although a recent study has documented the habituation and dishabituation paradigm of boar pheromone (13), direct molecular evidence for detecting steroid pheromones in the MOS or VNO remains elusive. Therefore, detailed studies on the role of MOS/VNO in pig chemical communication are needed, using appropriate manipulations, to decipher the importance of chemosensory system(s) in pheromone signaling. This will eventually help to establish the functions of specific neurons and the subsequent olfactory cascade of pheromone signaling. Ultimately, advancements in this basic research would pave the way to understanding chemical communication in pigs. Moreover, due to large olfactory receptor families in pigs, dramatic or subtle adaptations may happen depending on the surrounding chemical environment (65). Therefore, behavioral studies should be carefully designed to decode the pheromone signaling and processing.

### Dependency on olfaction in young pigs

Baldwin and Cooper (66) confirmed that olfactory bulbectomy had no effect on the feeding behavior of young pigs. Similarly, bilateral olfactory bulb ablation in prepubertal boars had no evident effect on subsequent aggressive or mating behaviors, testicular function, or salivary pheromone content, with the only effect detected being a reduction in olfactory epithelium height (67). However, Morrow-Tesch and McGlone (68) evidenced that a lidocaine flush of the olfactory system of piglets eliminated the attachment of piglets to the nipple of the sows, underscoring the importance of olfaction in neonatal feeding. Salazar et al. (69) confirmed functional VNO and accessory olfactory bulbs at and before birth in pigs that help guide the piglets for feeding. In concert with the study of Morrow-Tesch and McGlone (68), maternal pheromones are suspected to be involved in guiding feeding behavior. Moreover, analysis of amniotic fluid, colostrum and milk revealed a transnatal olfactory continuity through sow maternal fluids, by the presence of both OBP and fatty acids. Thus, they participate to the recognition of the mother by the neonate piglet (70).

## Novel molecules yet to be tested with pig olfactory system

Recent studies have reported various molecules potentially involved in pig chemical communication. These compounds, in part, were shown to elicit behavioral changes in pigs. For instance, quinoline was found to elicit erected ears in sows during the assessment of sexual behavioral scores (11). Also, a rabbit pheromone, 2-methyl-2-butenal, was reported to modify the fighting and feeding behaviors of weaned piglets (71). Further, Devaraj et al. (72) reported 4-ethylphenol and 3-methylphenol in the urine of immune-stimulated pigs, which may have implications for pig behavior studies. Although technical advancements in analytical platforms resulted in the identification of newer compounds in various secretions of pigs, a pheromonal property of the compounds needs to be validated. Considering this scenario, the newer compounds should be tested with different binding proteins of nasal mucus and VNO of pigs (boars, sows and piglets) to ascertain their role in chemical communication, which may help determine the nature (pheromone or odorant) of the compounds. Further, analysis of the compounds with various binding proteins will also reveal the specific olfactory subsystem involved in the chemo-communication of pigs. We suggest studying molecules (odorants, putative pheromones, etc.,) with different proteins using *in silico* or other related approaches to decipher the role of newer compounds in pig chemical communication.

## Conclusion

The pig is an immensely important food animal that relies heavily on olfaction for reproduction, social interaction, survival and feeding. The reproduction of pigs, in part, is mediated through chemical communication. However, unlike rodents, the chemosensory systems and their related components have been sparsely investigated in pigs. Despite studies on the effect of pheromones at the behavioral level, deciphering the interaction between the pheromone and the cognate receptor of the olfactory system is less categorical and remains elusive in pigs. In order to decipher the chemical communication system of pigs, a complete understanding of pheromones, carriers or binding proteins, and the potential molecular action of pheromones in olfactory systems/subsystems are required. Given the knowledge gaps, advancements in basic research are needed in pigs to establish the roles of pheromones and to discriminate the general odorant and putative pheromones.

## Author contributions

DS and RK perceived the idea and drafted the first version of the manuscript. PN-L revised the first draft of the manuscript. JA, SA, and GA critically revised the manuscript and drafted the final version. All authors contributed to the article and approved the submitted version.

## Conflict of interest

The authors declare that the review was written in the absence of any commercial or financial relationships that could be construed as a potential conflict of interest.

## Publisher's note

All claims expressed in this article are solely those of the authors and do not necessarily represent those of their affiliated organizations, or those of the publisher, the editors and the reviewers. Any product that may be evaluated in this article, or claim that may be made by its manufacturer, is not guaranteed or endorsed by the publisher.
